# Supplementary material for: Modeling of Personalized Treatments in Colon Cancer Based on Preclinical Genomic and Drug Sensitivity Data
Source: Cancers (Basel). 2021 Nov 30;13(23):6018. doi: 10.3390/cancers13236018 (PMC8656546; doi:10.3390/cancers13236018)
Supplement: Supplementary file 1 [file cancers-13-06018-s001.zip › cancers-1477565-supplementary.pdf]

# Supplement Figure S1: Subgroup I - MSI hypermutated

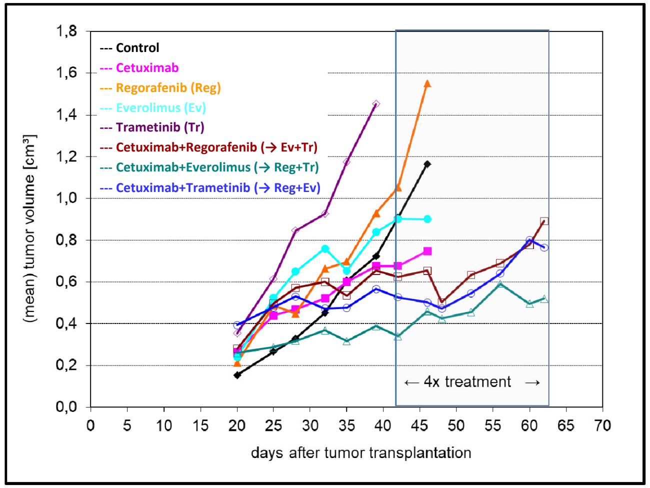


Co11476-299

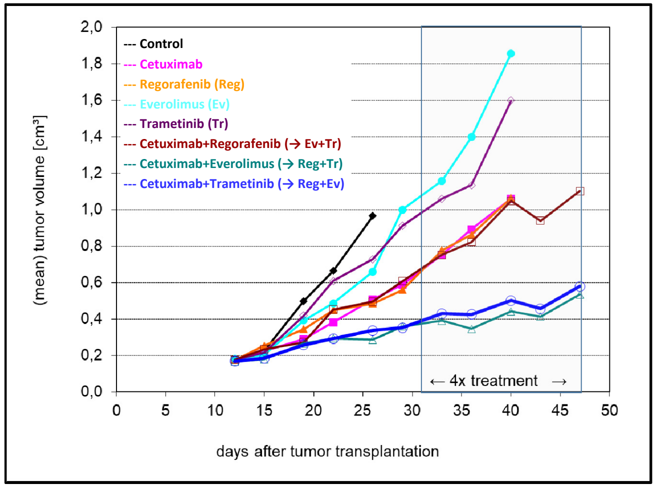


Co11309-278

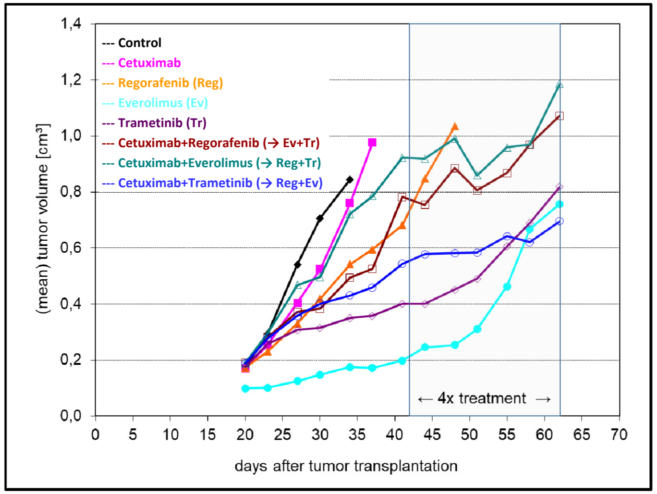


Co10039-106

with corresponding body weight graph

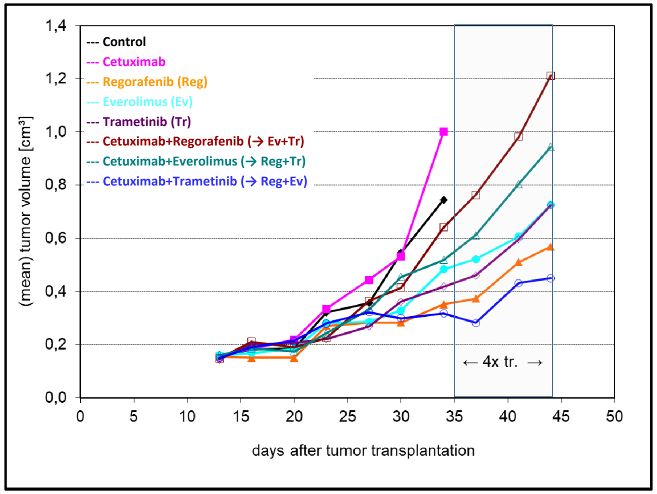


Co11203-261

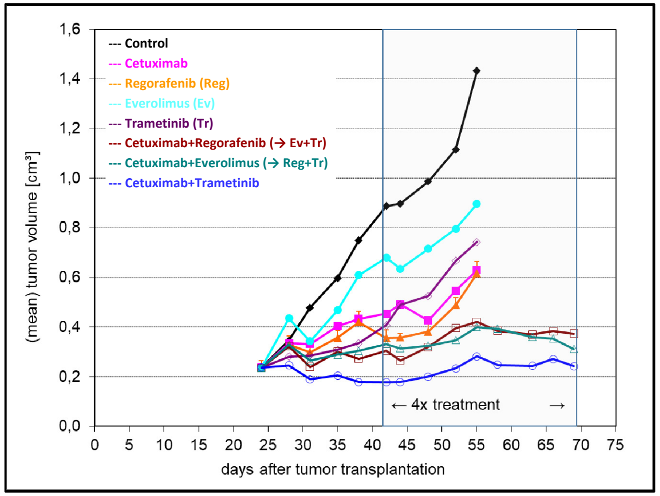


Co11672-327

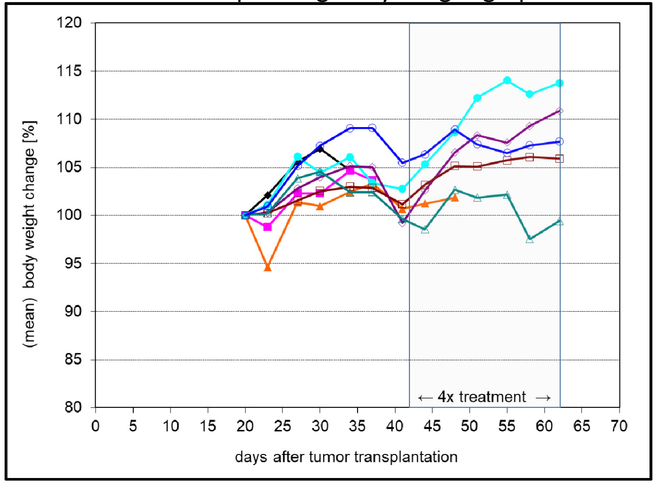

# Supplement Figure S2: Subgroup II - MSS BRAF mutated

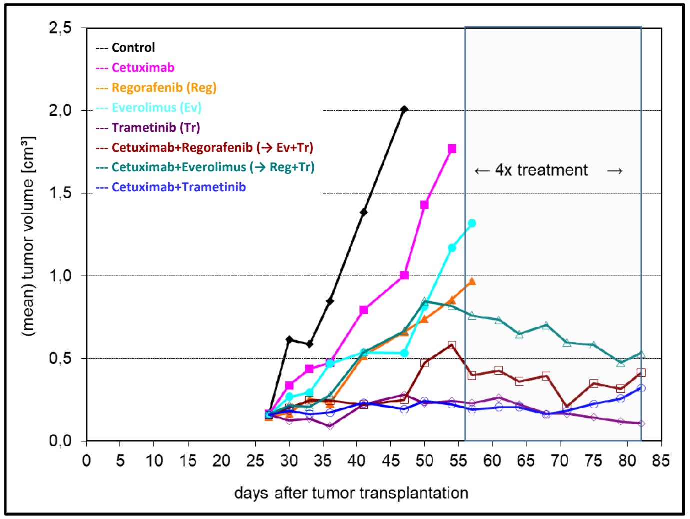


Co10786-181

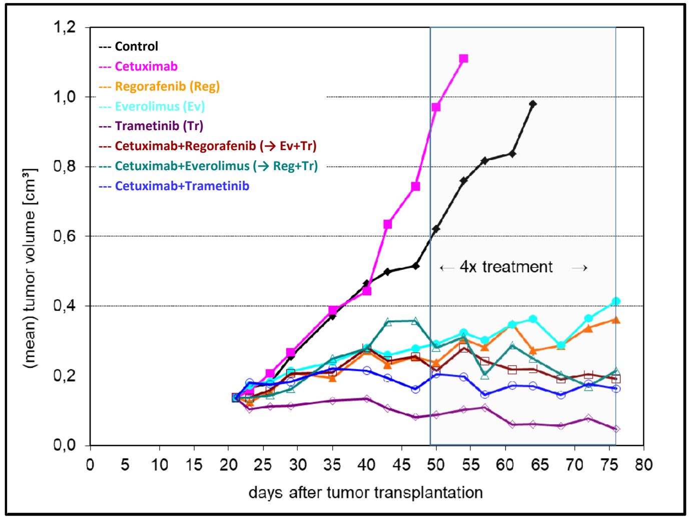


Co10979-212

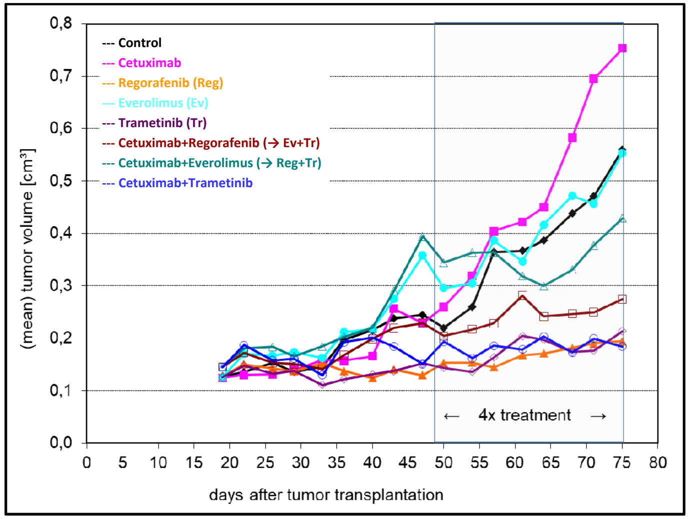


Co10629D-150

with corresponding body weight graph

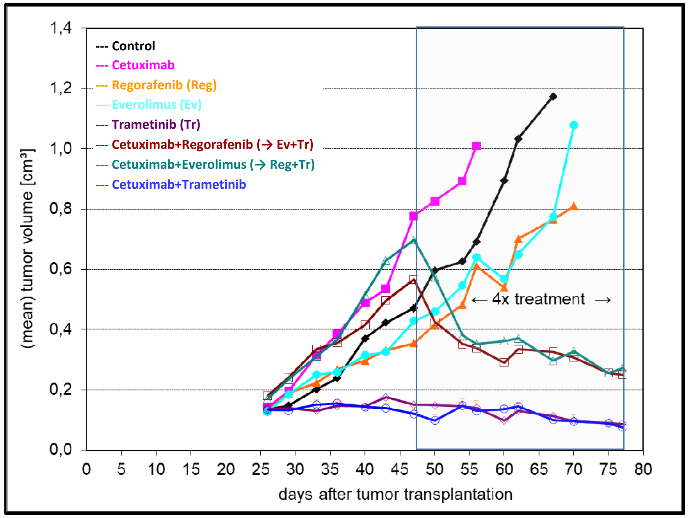


Co11388-289

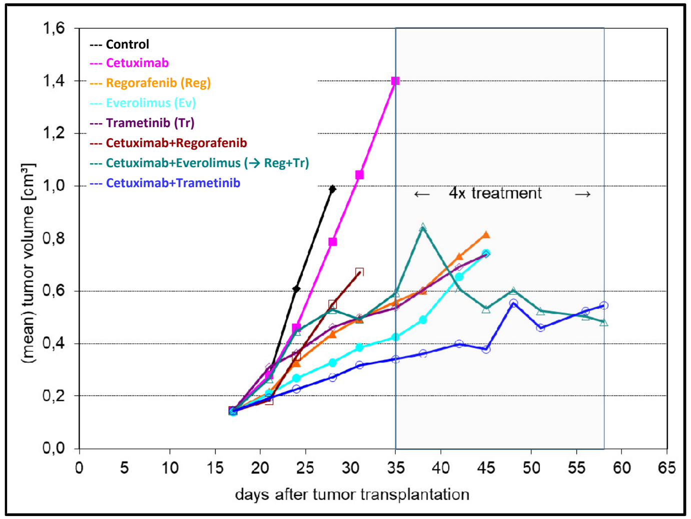


Co11336-283

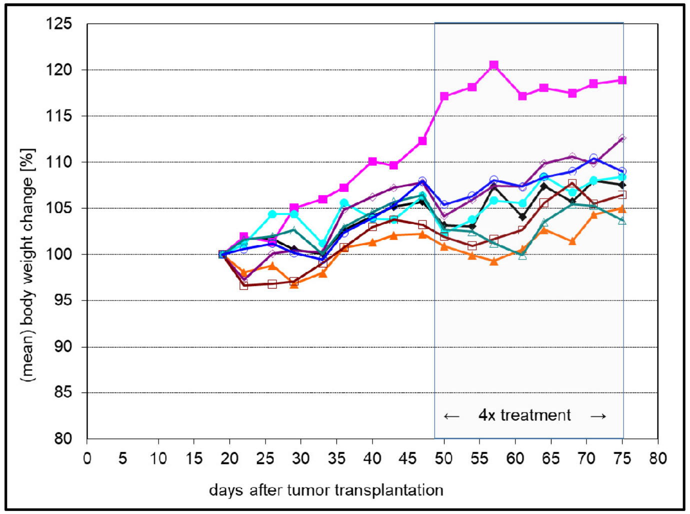

# Supplement Figure S3: Subgroup III - MSS KRAS and BRAF wild type

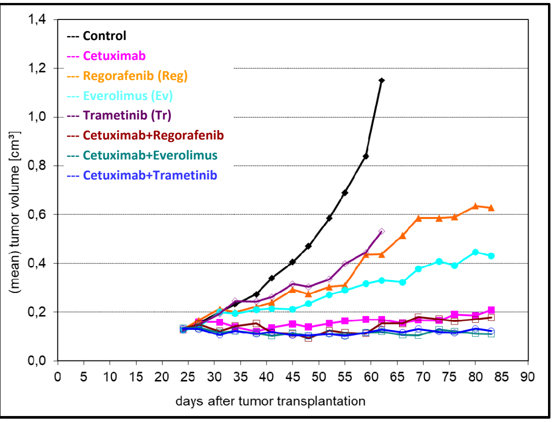


Co11003-216

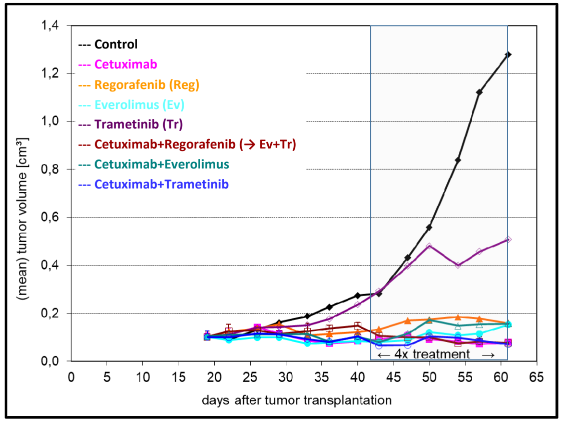


Co10567-139

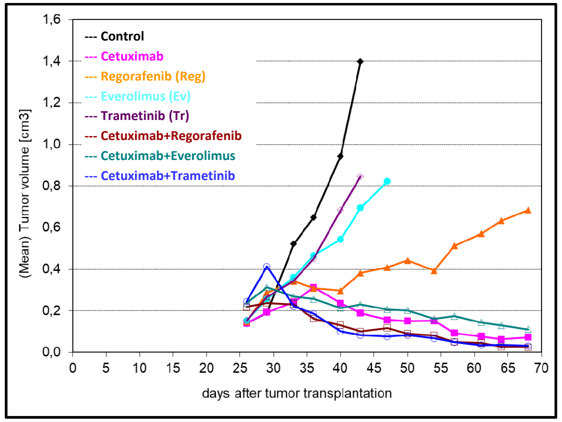


Co10389-116

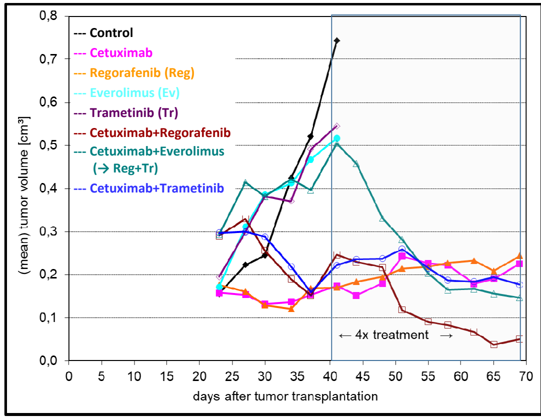


Co11291-273

with corresponding body weight graph

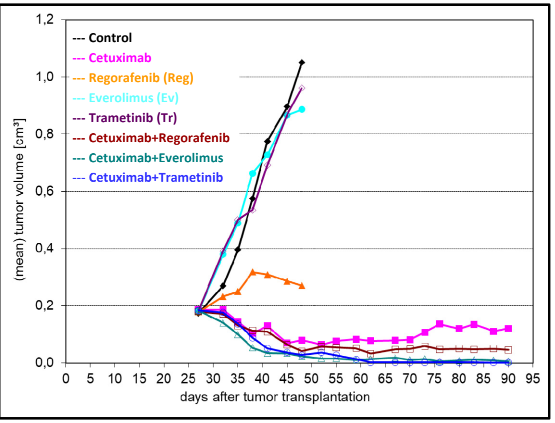


Co11192-259

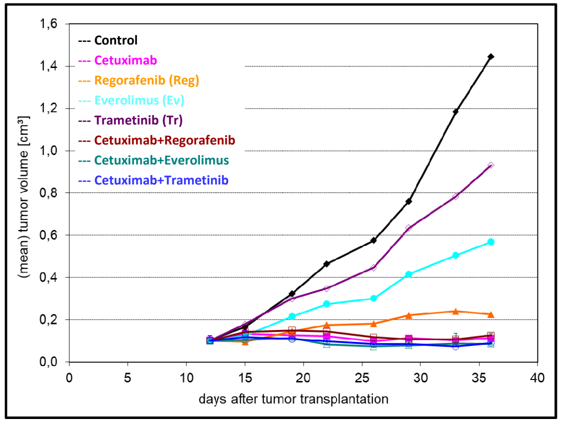


Co11246-208

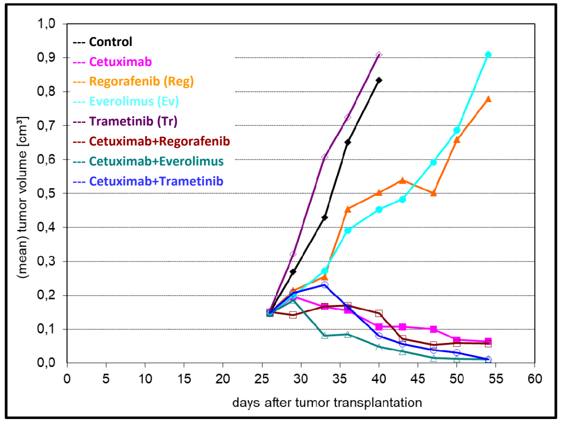


Co10849-191

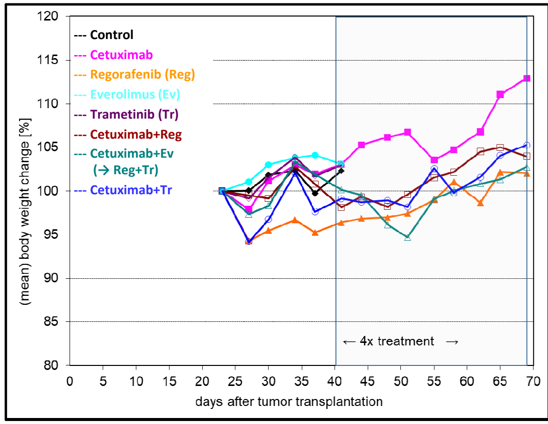

# Supplement Figure S4: Subgroup IV  -  MSS KRAS mutated

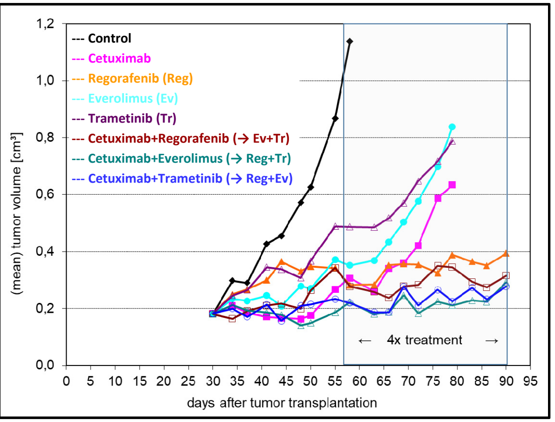

Co11319-280

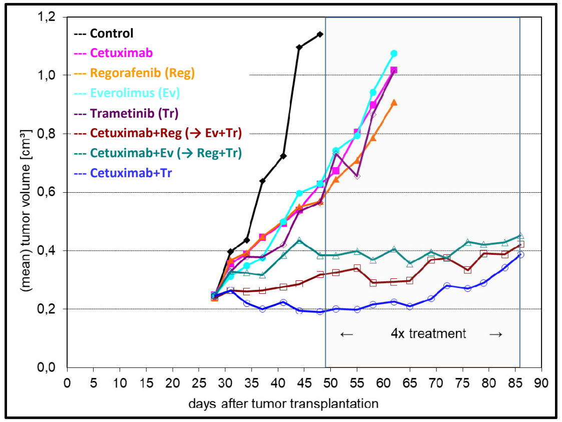

Co10748-171

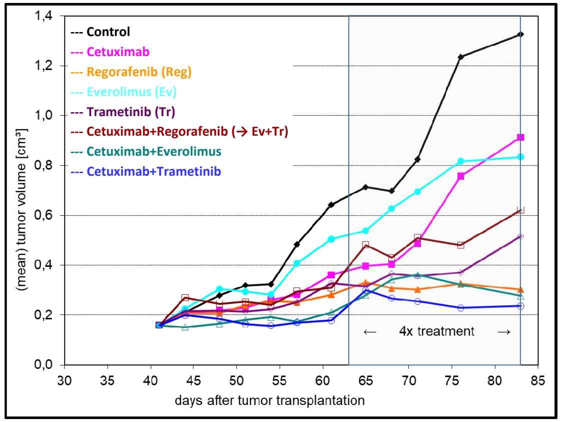

Co10501-118

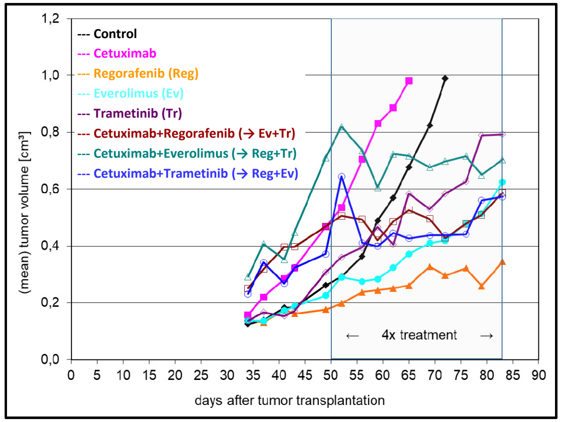

Co11300-277

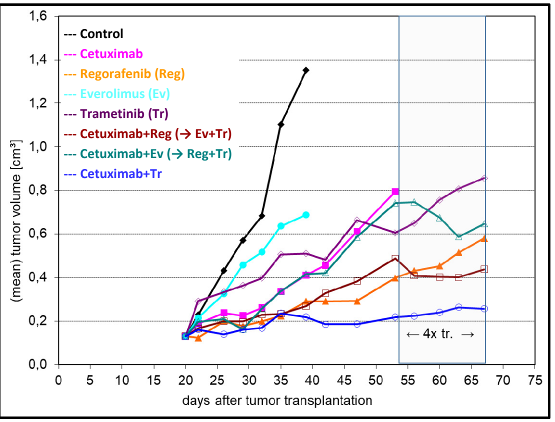

Co10803-183

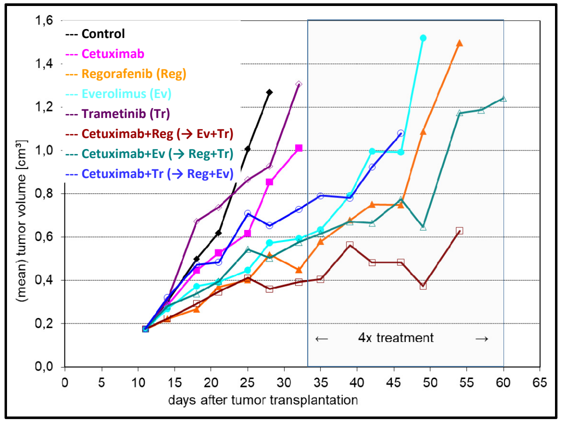

Co11993-352

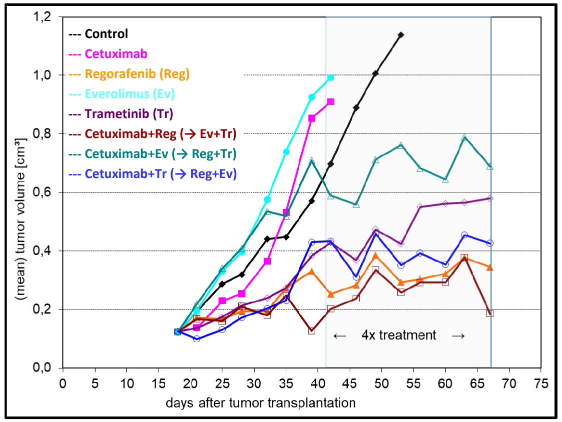

Co10412-128

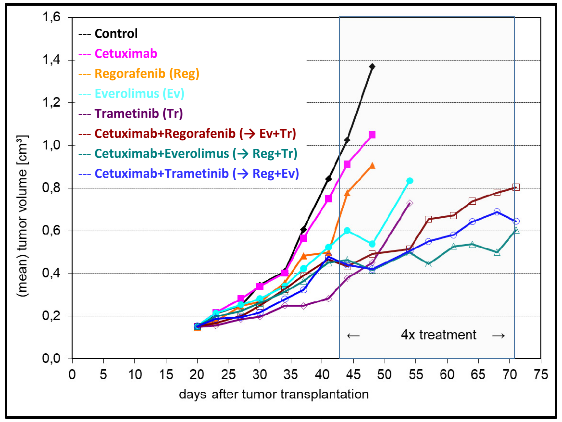

Co10348-114
